# Psychophysical Measures of Sensitivity to Facial Expression of Emotion

**DOI:** 10.3389/fpsyg.2013.00063

**Published:** 2013-02-20

**Authors:** Michelle Marneweck, Andrea Loftus, Geoff Hammond

**Affiliations:** ^1^Department of Psychology, The University of Western AustraliaPerth, WA, Australia; ^2^Department of Psychology, Curtin UniversityPerth, WA, Australia

**Keywords:** emotion perception, psychophysical methods, emotion discrimination, facial expression, emotional intensity

## Abstract

We report the development of two simple, objective, psychophysical measures of the ability to discriminate facial expressions of emotion that vary in intensity from a neutral facial expression and to discriminate between varying intensities of emotional facial expression. The stimuli were created by morphing photographs of models expressing four basic emotions, anger, disgust, happiness, and sadness with neutral expressions. Psychometric functions were obtained for 15 healthy young adults using the Method of Constant Stimuli with a two-interval forced-choice procedure. Individual data points were fitted by Quick functions for each task and each emotion, allowing estimates of absolute thresholds and slopes. The tasks give objective and sensitive measures of the basic perceptual abilities required for perceiving and interpreting emotional facial expressions.

## Introduction

The ability to perceive the facial expressions of emotion of others is central to the regulation of social behavior. Emotion perception has been studied in different populations including children (Gao and Maurer, 2009), young adult (Elfenbein and Ambady, 2002), and the aging (Sullivan and Ruffman, 2004), and in both healthy and clinical populations (Montagne et al., 2007; Assogna et al., 2008; Hippolyte et al., 2008; Hofer et al., 2009; Harms et al., 2010). Much of this research has focused on the ability to recognize facial expressions of emotion, usually assessed as the ability to identify specific emotions by name (retrieved either from memory or a list of names) or to distinguish different expressions of emotion (Calder et al., 2000; Matsumoto et al., 2000; Clark et al., 2008; Young and Hugenberg, 2010; Bell et al., 2011). The Ekman 60 Faces Test (Young et al., 2002) exemplifies a recognition test that requires participants to select from a list of six basic emotions the emotion that best describes the facial expression shown. The stimuli used in identification studies generally depict full-blown emotional facial expressions selected from validated stimulus sets (Matsumoto and Ekman, 1988; Tottenham et al., 2009). In everyday life, however, emotions are generally expressed with graded intensity. There has been some interest in the ability to identify graded intensities of facial expressions of emotion with rating scales (Matsumoto et al., 2000; Dujardin et al., 2004). There has also been interest in using dynamic morphed stimuli (from an emotional face to a neutral face, and from one emotion to another) to measure the point at which an emotion becomes apparent from a neutral expression and at which a change in emotion is detected (Niedenthal et al., 2000, 2001; Montagne et al., 2007; Fiorentini and Viviani, 2011; Sacharin et al., 2012). The ability to distinguish between confusable expressions has been assessed with tests such as the Emotion Hexagon Test (Young et al., 2002), which requires participants to name the emotional term that best describes images composed of graded blends of two confusable emotional expressions (such as happiness and surprise and disgust and anger).

Despite the interest in the ability to identify and to distinguish facial expressions of emotion, the basic perceptual abilities that may assist the more complex processes of identifying a specific emotion by name and distinguishing between emotions remain less explored. Measurements of the perceptual processes target the ability to discriminate speedily the visual properties of facial expressions that indicate the emotion and its intensity (Adolphs, 2002). In contrast, the more complex processes place demands on verbal processes, including vocabulary (Adolphs, 2002), and on working memory (Phillips et al., 2008). There are currently no sensitive, psychophysical measures of the fundamental perceptual abilities of discriminating emotional from neutral facial expressions and discriminating varying intensities of facial expressions of emotion. Psychophysical methods offer objective, sensitive, and efficient measures of perceptual processes that are relatively free from response criterion effects. The aim of this study was to determine the usefulness of psychophysical measures of the ability to discriminate emotional from neutral expressions and to discriminate between graded intensities of emotional expression for four commonly expressed emotions, anger, disgust, happiness, and sadness. The emphasis of the paper is on the demonstration of the method and the usefulness of the general approach.

## Methods

### Participants

Fifteen healthy young adult volunteers (nine females) with no reported neurological impairments were tested. Their ages ranged from 22 to 27 years. Two other volunteers participated in a preliminary phase to select the stimuli. The procedures were approved by the Institutional Ethics Committee and all participants gave written informed consent.

### Materials and procedures

#### Development of the stimulus set

**Figure 1:**
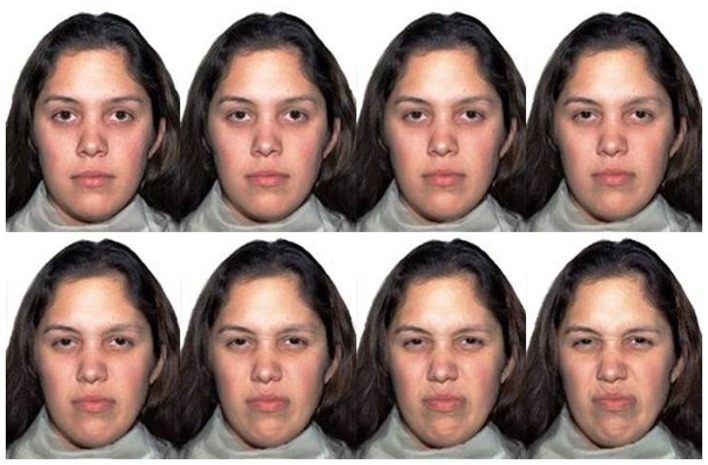
**Morphed stimuli of a neutral expression (defined as 0% expressivity) and a full-blown expression of disgust (defined as 100% expressivity)**. The eight images vary in 10% equally spaced increments starting from 10 to 80% expressivity of disgust.

We selected colored photographs of models expressing emotions from a validated set (the NimStim Face Stimulus Set; Tottenham et al., 2009) for each of four basic emotions, anger, disgust, happiness, and sadness. The six Caucasian models (three male, three female) that produced the highest agreement of their intended expressions in a validation study (Tottenham et al., 2009) were used. Neutral expressions of the models (rated as an expressive intensity of zero) were morphed with their full-blown emotional expressions (rated as an expressive intensity of 100%) in steps of 5% with Norrkross MorphX software (Wennerberg, 1997) to create graded intensities of expression for each emotion. The Norrkross software is a freeware, open-source program that allows morphing of two photographic images creating a prototypical facial image from exemplars using a sophisticated morphing algorithm that implements the principles described by Benson and Perrett, 1993, as cited by Pearson and Adamson, 2004). The software is widely used in research (Pearson and Adamson, 2004; Liu and Jagadeesh, 2008; Akrami et al., 2009; Vida and Mondloch, 2009; Ishikawa and Mogi, 2011). Similar to the work of Pearson and Adamson (2004), an average of 75 key points were allocated to identify points of similarity between the faces, with more points assigned around areas of greater change with increasing emotional intensity, such as around the pupils, eyelids, eyebrows, lips, and nose. The software also allows for the points to be connected with Bezier curves to define the warping region for further precision (Pearson and Adamson, 2004). Expressions of anger and happiness, which are typically expressed with an open mouth, were morphed with open mouth neutral expressions, and expressions of disgust and sadness, which are typically expressed with a closed-mouth, were morphed with closed-mouth neutral expressions. Two models were selected for each emotion to ensure that judgments were not made only of the specific features of a single model. Figure 1 shows an example of the morphed stimuli from 10 to 80% expressivity of disgust.

#### Experimental procedure

The ability to discriminate an emotional from a neutral expression and to discriminate between different intensities of expression of the same emotion was measured with two tasks (with two variants of the second) using a two-interval forced-choice layout with the Method of Constant Stimuli. On each trial, two faces of the same model were presented successively on a computer screen for 200 ms with a 200-ms blank inter-stimulus interval. The 200-ms inter-stimulus interval was sufficiently long to prevent transformational apparent motion from the first to the second image (Kawahara et al., 1996). The face stimuli were 68 mm high and 54 mm wide subtending visual angles of 6.6° and 5.2° at a viewing distance of 590 mm. Response time was unlimited and followed by a 1-s blank window (no feedback given) before the next trial commenced.

In the task that required discrimination of a neutral from an emotional expression, the face with the neutral expression appeared randomly in either the first or the second interval and a face expressing one of seven levels of intensity of the tested emotion appeared in the other interval. The seven intensity levels ranged from 5 to 35% of the full-blown expression in equally spaced increments. The stimulus levels and range were chosen using pilot data from two participants to obtain unbiased and precise absolute thresholds (Swanson and Birch, 1992). On each trial, participants were required to signal which interval contained the face expressing the emotion by clicking either the left or right button on a mouse for the first or second interval respectively. The stimulus pairs were presented in randomized blocks of 14 trials (seven intensities × two models). There were 20 blocks resulting in a total of 280 trials, giving 40 trials for each intensity increment. The task was repeated for each of the four emotions with a 2-min break between each.

In the task that required discrimination between different intensities of the same emotion, two faces expressing different intensities were randomly assigned to the two intervals. The two facial expressions varied in five intensity steps from 5 to 25% in equally spaced increments, again chosen using pilot data (Swanson and Birch, 1992). Participants were required to signal which interval contained the face expressing the higher intensity by clicking either the left or right mouse button for the first or second observation interval respectively. Two variants of this task were run in the same session: one sampled expression intensities from a low-intensity range (from 10 to 50% of the full-blown expression) and the other from a high-intensity range (from 50 to 90% of the full-blown emotion). The stimuli used to define each intensity difference for each of these two sub-tasks are shown in Table 1; each intensity difference was defined by four different intensity pairs to establish generality of discriminating intensity differences across the range of intensities. The stimulus pairs were presented in randomized blocks of 40 trials, with one presentation of each of the four definitions of the five intensity differences (see Table 1) for each of two models in each block. There were five blocks for a total of 200 trials, giving 40 trials for each intensity difference.

**Table 1:** **Pairings of the different emotional intensities used to define each intensity difference for the task requiring discrimination of graded intensities for the low-intensity and high-intensity ranges**.

Graded intensity range	5%	10%	15%	20%	25%
**LOW-RANGE**					
Pair 1	10, 15	10, 20	10, 25	10, 30	10, 35
Pair 2	15, 20	15, 25	15, 30	15, 35	15, 40
Pair 3	20, 25	20, 30	20, 35	20, 40	20, 45
Pair 4	25, 30	25, 35	25, 40	25, 45	25, 50
**HIGH-RANGE**					
Pair 1	50, 55	50, 60	50, 65	50, 70	50, 75
Pair 2	55, 60	55, 65	55, 70	55, 75	55, 80
Pair 3	60, 65	60, 70	60, 75	60, 80	60, 85
Pair 4	65, 70	65, 75	65, 80	65, 85	65, 90

The two tasks were done in counterbalanced order in two separate testing sessions separated by at least 24 h. The presentation order of emotions within each task followed a Latin Square sequence and the order of the two sub-tasks was counterbalanced. Participants read standardized instructions before each task and were given five practice trials immediately before each task began.

#### Data analysis

Individual data obtained in each of the three determinations (discriminating emotional from neutral expressions, and discriminating different intensities of emotion in both the low and high-intensity range) for each of the four emotions (anger, disgust, happiness, and sadness) were fitted with Quick functions (Quick, 1974; Gilchrist et al., 2005) constrained to begin at 50%. The functions generally fitted the individual data well; the median *R*^2^ values for the four emotions ranged from 0.89 to 0.96 when discriminating emotional from neutral expressions, from 0.89 to 0.95 when discriminating different low-range intensities of emotion, and from 0.83 to 0.92 when discriminating different high-range intensities of emotion. The functions fitted to the individual and mean data for each of the three psychophysical tasks for each of the four emotions are shown in the figures. The absolute thresholds were taken as the intensity increment from neutral or the intensity differentiation that produced 75% correct performance. Summary descriptive statistics of thresholds and slopes are shown in the tables. Thresholds and slopes could not be obtained in 11 of the 180 individual determinations because the range of constant stimuli used did not capture a complete psychometric function or because the fit to the individual data points was poor. Participants were excluded from the statistical analyses of between-emotion effects on threshold and slope if one or more determinations were missing. Although the purpose of this report is to show the practical applicability of the method, and not the implications of the results themselves, we report one-way repeated-measures analyses of variance on thresholds and slopes for those participants with complete data sets. The Greenhouse Geisser correction was applied to the data where the sphericity assumption was violated. The statistical analyses serve to show the sensitivity of the measures to the emotional expression tested and are not used to make any claims about the nature of processing expressions of the different emotions.

## Results

### Discriminating emotional from neutral expressions

**Figure 2:**
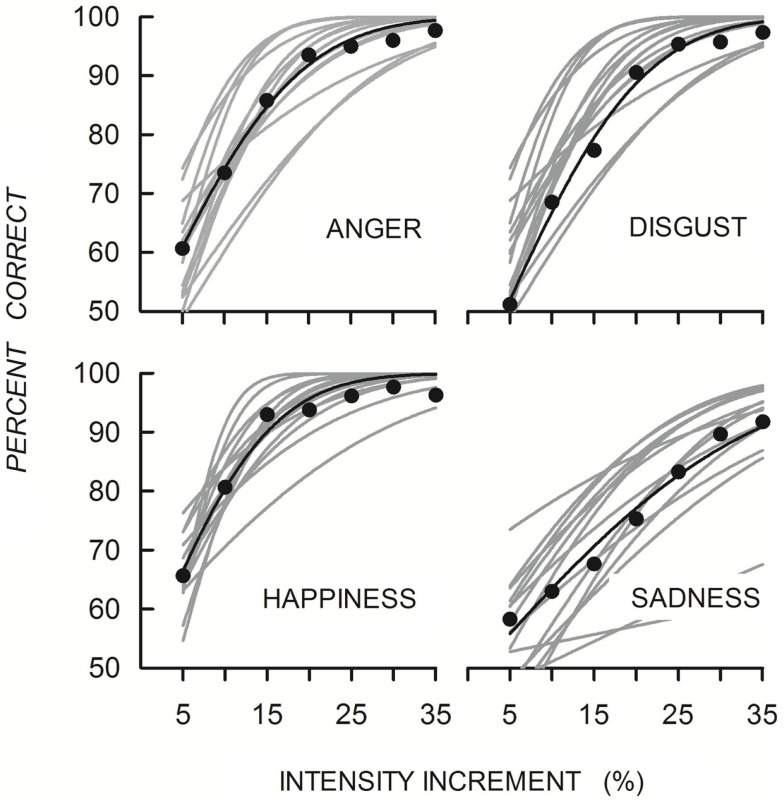
**Quick functions fitted to individual (gray lines) and mean accuracy (black symbols and lines) when discriminating emotion from neutral expressions for each of the four emotions**.

Figure 2 shows the functions fitted to individual and mean data points. All functions increased monotonically with increasing differentiation of the expressive from the neutral face for each of the four emotions, with individual variation in level and slope. Mean absolute thresholds and slopes with the sample sizes for each are shown in Table 2. Thresholds ranged from about 8% to about 17% and were lowest for expressions of happiness and highest for expressions of sadness. Analysis of 11 thresholds of complete data sets showed a significant main effect of Emotion [*F*(3, 30) = 16.10, *p* < 0.001, η $\eta_{p}^{2}$ = 0.62]. There was no effect of emotion for slopes of 11 data sets [*F*(1.49, 14.86) = 0.90, *p* > 0.05, η $\eta_{p}^{2}$ = 0.08].

**Table 2:** **Mean absolute thresholds and slopes for discriminating emotional from neutral expressions for each of the four emotions**.

	Anger	Disgust	Happiness	Sadness
Threshold	10.5 (3.6)	13.6 (3.0)	7.8 (1.9)	16.9 (5.0)
Slope	2.2 (2.0)	2.4 (1.0)	1.5 (0.6)	1.9 (0.8)
*N*	15	14	15	12

*The *N* for each emotion is shown and the standard deviations are in parentheses*.

*Satisfactory curve fits were not obtained for four participants, one in the Disgust condition and three in the Sadness condition*.

### Discriminating different intensities of emotional expression

**Figure 3:**
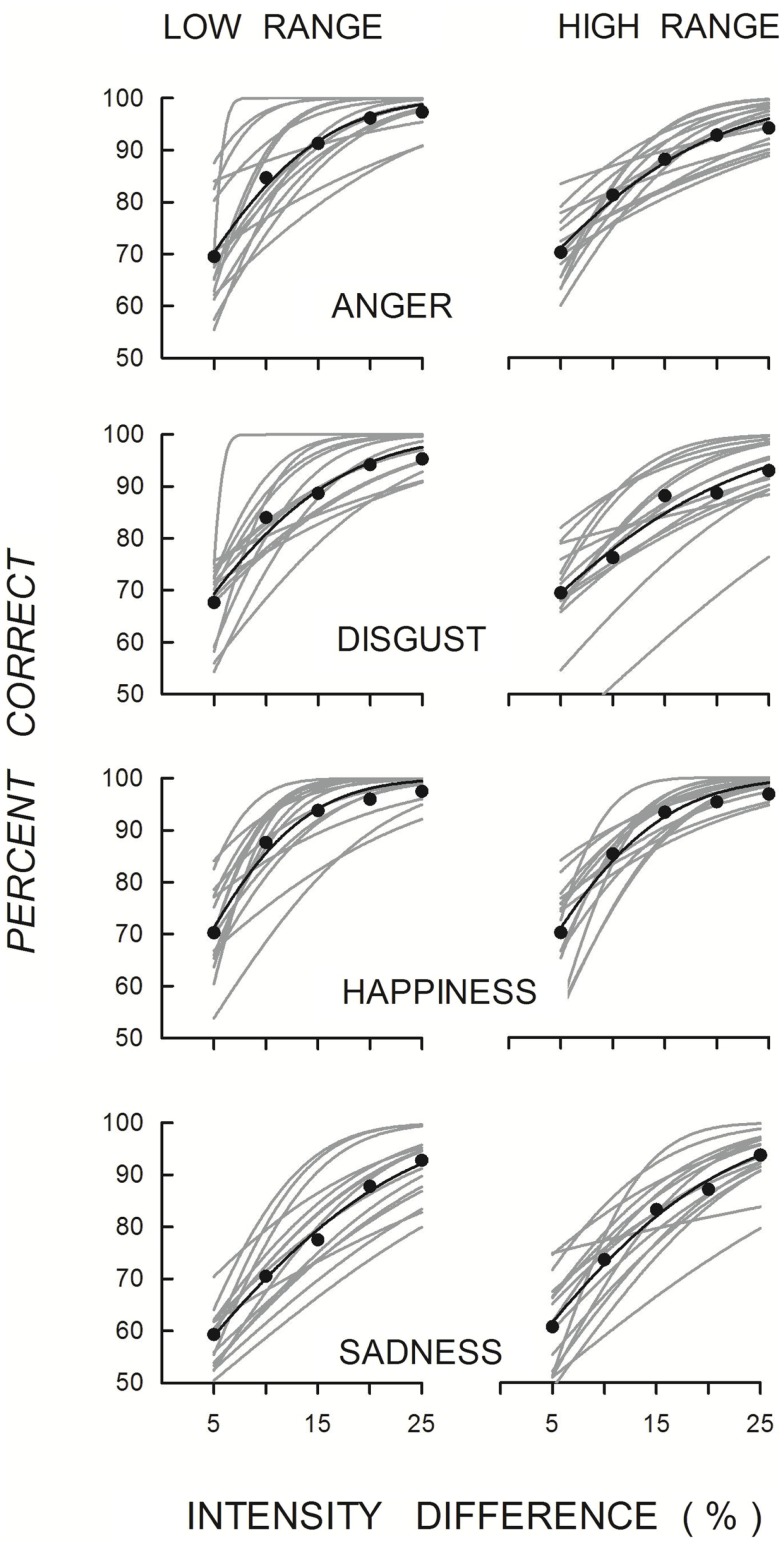
**Quick functions fitted to individual (gray lines) and mean accuracy (black symbols and lines) when discriminating different intensities of emotional expression for each intensity range (left panels: low-intensity range; right panels: high-intensity range) for each of the four emotions**.

Figure 3 shows the functions fitted to individual and mean data points. The mean functions increased with increasing intensity differentiation of the emotional expressions in both intensity ranges, with individual variation again evident. Mean thresholds and slopes derived from the fitted functions with the sample sizes for each are shown in Table 3. Absolute thresholds for each emotional expression were similar in both intensity ranges, and, consistent with the previous measure, were lowest for discriminating different intensities of happiness and highest for discriminating different intensities of sadness. The main effect of Emotion was significant from analyses of 12 data sets for the low-intensity range [*F*(3, 33) = 22.70, *p* < 0.001, η $\eta_{p}^{2}$ = 0.67] and 11 data sets for high-intensity range [*F*(1.39, 13.87) = 6.88, *p* = 0.01, η $\eta_{p}^{2}$ = 0.41]. The slopes for each emotional expression were similar in the two intensity ranges, and were similar for the emotional expressions in each of the intensity ranges [low-intensity range, *F*(3, 33) = 0.29, *p* > 0.05, η _p^2 = 0.02; high-intensity range, *F*(1.35, 13.50) = 2.37, *p* = 0.14, η $\eta_{p}^{2}$ = 0.19].

**Table 3:** **Mean absolute thresholds and slopes for discriminating between varying intensities of the four emotions for the low- and high-intensity ranges**.

	Anger	Disgust	Happiness	Sadness
**LOW-RANGE**				
Threshold	7.4 (2.8)	7.3 (2.3)	6.3 (2.5)	12.7 (4.1)
Slope	1.5 (0.5)	1.5 (1.0)	1.5 (0.6)	1.7 (0.4)
*N*	13	15	15	14
**HIGH-RANGE**				
Threshold	7.1 (1.9)	8.9 (5.5)	6.1 (2.3)	11.4 (4.3)
Slope	1.0 (0.4)	1.2 (0.5)	1.3 (0.7)	1.9 (1.5)
*N*	14	13	15	14

*The *N* for each emotion in each range is shown and the standard deviations are in parentheses*.

*Satisfactory curve fits were not obtained for three participants in the low-range variant of the task, two in the Anger condition and one in the Sadness condition and for four participants in the high-range variant of the task, one in the Anger condition, two in the Disgust condition, and one in the Sadness condition*.

## Discussion

The results show that the psychophysical method described here is suitable for measuring sensitivity to facial expressions of four basic emotions in healthy young adults. The mean data in each of the measures were well fitted by the Quick functions, giving estimates of threshold and slope for each expression in each of the three tasks. The data also reveal individual differences in discrimination performance in the sample of healthy young adults, presumably reflecting in part individual differences in sensitivity to gradations in intensity of facial expressions of emotion. Psychometric functions could not be fitted in some cases in which performance was at or above 75% correct at the smallest constant stimulus value. This issue is easily addressed in future work by increasing the range of constant stimulus values by selecting them to form a multiplicative scale, with each value a multiple of the previous value, rather than the additive scale used here. Selecting the constant stimuli in this way will allow the full range of psychophysical performance to be captured. Future work could also merge the low- and high-range variants for further efficiency, given similar threshold and slope values found for both variants. We suggest selecting intensity pairs across the entire intensity range to define each intensity difference.

The method offers four major advantages over commonly used methods of sensitivity to emotional expression such as identification and rating the perceived intensity of an expressed facial emotion. First, the forced-choice methodology is relatively free from response biases and subjective criterion, and therefore gives an objective measure of sensitivity that is not matched by subjective measures. Second, forced-choice methodology gives sensitive measures of the ability to discriminate facial expressions of emotion. The sensitivity to small variations in the intensity with which an emotion is expressed is shown by the small absolute thresholds, which ranged from about 7% to about 17% (with a median of 9%) in the different measures. This sensitivity makes the method capable of detecting small changes in sensitivity to emotional expression that might result from an experimental manipulation or that might emerge with healthy aging or the progression of a neurological disorder. Third, the method is broadly applicable to the expression of different emotional states. Although broadly applicable, the method’s sensitivity revealed differences in the ability to detect changes in different emotional expressions, with consistently smaller absolute thresholds for detecting changes in happiness than sadness, and intermediate thresholds for disgust and anger. Emotional expressions vary in the visual range of expressivity, so the magnitude of a 5% change will vary depending on the emotion. Changes in an emotion such as happiness, which is typically expressed with an open mouth and with extensive changes in facial features, will be discriminated with a smaller percentage change than an emotion that is more subtly expressed, such as sadness, which is typically expressed with a closed-mouth and less extensive changes in facial features. The variations in ability to detect changes for different emotional expressions are consistent with previous research on more complex processes showing that expressions of positive emotions are easier to identify than negative emotions (Elfenbein and Ambady, 2002). Fourth, the method is efficient, which is an important factor when testing aged or clinical populations. A psychometric function with 40 observations at each of seven points can be obtained in about 12 min. The method could be made even more efficient by estimating threshold level with an adaptive procedure, in which the stimuli are changed contingent on the observer’s response, in place of the constant stimulus method used here. Fifth, the method is simple to administer and easily understood by participants. Although the results reported here are from a select sample of young, healthy, educated adults, our research in progress shows that the method is equally applicable to samples of patients with Parkinson’s disease and their age-matched controls, and so encourages its use in other atypical and healthy aging populations. It has been shown that emotion recognition is modulated by the mood of the perceiver (Niedenthal et al., 2000, 2001). It remains to be determined if the processes of emotion detection and discrimination measured by the methods described here are also susceptible to mood.

The basic perceptual abilities measured by the methods reported here, the ability to discriminate an emotional from a neutral expression, and the ability to differentiate between two different levels of expression of the same emotion, may assist or work in concert with more complex social decision making and behavior. These measures, therefore, allow the contribution of lower-order perceptual determinants of higher-order disorders of emotional judgment to be detected.

## Conflict of Interest Statement

The authors declare that the research was conducted in the absence of any commercial or financial relationships that could be construed as a potential conflict of interest.

## References

[B1] Adolphs R. (2002). Recognizing emotion from facial expressions: psychological and neurological mechanisms. Behav. Cogn. Neurosci. Rev. 1, 21–61 10.1177/1534582302001001003 17715585

[B2] Akrami A. Liu Y. Treves A. Jagadeesh B. (2009). Converging neuronal activity in inferior temporal cortex during the classification of morphed stimuli. Cereb. Cortex 19, 760–776 10.1093/cercor/bhn125 18669590 PMC2651479

[B3] Assogna F. Pontieri F. E. Caltagirone C. Spalletta G. (2008). The recognition of facial emotion expressions in Parkinson’s disease. Eur. Neuropsychopharmacol. 18, 835–848 10.1016/j.euroneuro.2008.07.004 18707851

[B4] Bell C. Bourke C. Colhoun H. Carter F. Frampton C. Porter R. (2011). The misclassification of facial expressions in generalized social phobia. J. Anxiety Disord. 25, 278–283 10.1016/j.janxdis.2010.10.001 21041060

[B5] Benson P. J. Perrett D. I. (1993). Extracting prototypical facial images from exemplars. Perception 22, 257–262 10.1068/p220257 8316513

[B6] Calder A. J. Keane J. Cole J. Campbell R. Young A. W. (2000). Facial expression recognition by people with Mobius syndrome. Cogn. Neuropsychol. 17, 73–87 10.1080/026432900380490 20945172

[B7] Clark U. S. Neargarder S. Cronin-Golomb A. (2008). Specific impairments in the recognition of emotional facial expressions in Parkinson’s disease. Neuropsychologia 46, 2300–2309 10.1016/j.neuropsychologia.2008.03.014 18485422 PMC2491661

[B8] Dujardin K. Blairy S. Defebvre L. Duhem S. Hess U. Destée A. (2004). Deficits in decoding emotional facial expressions in Parkinson’s disease. Neuropsychologia 42, 239–250 10.1016/S0028-3932(03)00154-4 14644109

[B9] Elfenbein H. A. Ambady N. (2002). On the universality and cultural specificity of emotion recognition: a meta-analysis. Psychol. Bull. 128, 203–235 10.1037/0033-2909.128.2.203 11931516

[B10] Fiorentini C. Viviani P. (2011). Is there a dynamic advantage for facial expressions? J. Vis. 11, 1–15, 17. 10.1167/11.3.1 21427208

[B11] Gao X. Maurer D. (2009). Influence of intensity on children’s sensitivity to happy, sad, and fearful facial expressions. J. Exp. Child. Psychol. 102, 503–521 10.1016/j.jecp.2008.11.002 19124135

[B12] Gilchrist J. M. Jerwood D. Ismaiel H. S. (2005). Comparing and unifying slope estimates across psychometric function models. Percept. Psychophys. 67, 1289–1303 10.3758/BF03193560 16502849

[B13] Harms M. B. Martin A. Wallace G. L. (2010). Facial emotion recognition in autism spectrum disorders: a review of behavioral and neuroimaging studies. Neuropsychol. Rev. 20, 1–33 10.1007/s11065-010-9138-6 20809200

[B14] Hippolyte L. Barisnikov K. Van der Linden M. (2008). Face processing and facial emotion recognition in adults with Down syndrome. Am. J. Ment. Retard. 113, 292–306 10.1352/0895-8017(2008)113[292:FPAFER]2.0.CO;2 18564889

[B15] Hofer A. Benecke C. Edlinger M. Huber R. Kemmler G. Rettenbacher M. A. (2009). Facial emotion recognition and its relationship to symptomatic, subjective, and functional outcomes in outpatients with chronic schizophrenia. Eur. Psychiatry 24, 27–32 10.1016/S0924-9338(09)70260-3 18774270

[B16] Ishikawa T. Mogi K. (2011). Visual one-shot learning as an ‘anti-camouflage device’: a novel morphing paradigm. Cogn. Neurodyn. 5, 231–239 10.1007/s11571-011-9171-z 22942913 PMC3179546

[B17] Kawahara J. Yokosawa K. Nishida S. Sato T. (1996). Illusory line motion in visual search: attentional facilitation or apparent motion? Perception 25, 901–920 10.1068/p250901 8938004

[B18] Liu Y. Jagadeesh B. (2008). Neural selectivity in anterior inferotemporal cortex for morphed photographic images during behavioral classification or fixation. J. Neurophysiol. 100, 966–982 10.1152/jn.01354.2007 18234975 PMC2652138

[B19] Matsumoto D. Ekman P. (1988). Japanese and Caucasian Facial Expressions of Emotion and Neutral Faces (JACFEE and JACNeuF). San Francisco, CA: Human Interaction Laboratory, University of California

[B20] Matsumoto D. LeRoux J. Wilson-Cohn C. Raroque J. Kooken K. Ekman P. (2000). A new test to measure emotion recognition ability: Matsumoto and Ekman’s Japanese and Caucasian brief affect recognition test (JACBART). J. Nonverbal Behav. 24, 179–209 10.1023/A:1006668120583

[B21] Montagne B. Kessels R. P. C. De Haan E. H. F. Perrett D. I. (2007). The emotion recognition task: a paradigm to measure the perception of facial emotional expressions at different intensities. Percept. Mot. Skills 104, 589–598 10.2466/pms.104.2.589-598 17566449

[B22] Niedenthal P. M. Brauer M. Halberstadt J. B. Innes-Ker A. H. (2001). When did her smile drop? Facial mimicry and the influences of emotional state on the detection of change in emotional expression. Cogn. Emot. 15, 853–864 10.1080/02699930143000194

[B23] Niedenthal P. M. Halberstadt J. B. Margolin J. Innes-Ker A. H. (2000). Emotional state and the detection of change in facial expression of emotion. Eur. J. Soc. Psychol. 30, 211–222 10.1002/(SICI)1099-0992(200003/04)30:2<211::AID-EJSP988>3.0.CO;2-3

[B24] Pearson D. C. Adamson P. A. (2004). The ideal nasal profile: rhinoplasty patients vs the general public. Arch. Facial Plast. Surg. 6, 257–262 10.1001/archfaci.6.4.257 15262721

[B25] Phillips L. H. Channon S. Tunstall M. Hedenstrom A. Lyons K. (2008). The role of working memory in decoding emotions. Emotion 8, 184–191 10.1037/1528-3542.8.2.184 18410192

[B26] Quick R. (1974). A vector-magnitude model of contrast detection. Kybernetik 16, 65–67 10.1007/BF00271628 4453110

[B27] Sacharin V. Sander D. Scherer K. R. (2012). The perception of changing emotion expressions. Cogn. Emot. 26, 1273–1300 10.1080/02699931.2012.656583 22550942

[B28] Sullivan S. Ruffman T. (2004). Emotion recognition deficits in the elderly. Int. J. Neurosci. 114, 403–432 10.1080/00207450490270901 14754664

[B29] Swanson W. H. Birch E. E. (1992). Extracting thresholds from noisy psychophysical data. Atten. Percept. Psychophys. 51, 409–422 10.3758/BF03211637 1594431

[B30] Tottenham N. Tanaka J. W. Leon A. C. McCarry T. Nurse M. Hare T. A. (2009). The NimStim set of facial expressions: judgments from untrained research participants. Psychiatry Res. 168, 242–249 10.1016/j.psychres.2008.05.006 19564050 PMC3474329

[B31] Vida M. D. Mondloch C. J. (2009). Children’s representations of facial expression and identity: identity-contingent expression after affects. J. Exp. Child. Psychol. 104, 326–345 10.1016/j.jecp.2009.06.003 19632689

[B32] Wennerberg M. (1997). Norrkross MorphX(Version 2.9.5) [Computer Software]. Available at: http://www.norrkross.com/software/morphx/morphx.php

[B33] Young A. W. Perrett D. I. Calder A. J. Sprengelmeyer R. Ekman P. (2002). Facial Expressions of Emotion: Stimuli and Tests (FEEST) [Computer Software]. Bury St Edmunds: Thames Valley Test Company

[B34] Young S. G. Hugenberg K. (2010). Mere social categorization modulates identification of facial expressions of emotion. J. Pers. Soc. Psychol. 99, 964–977 10.1037/a0020400 20919774

